# Superior Vena Cava Syndrome Secondary to the Largest Reported Thoracic Hydatid Disease

**DOI:** 10.7759/cureus.28861

**Published:** 2022-09-06

**Authors:** Ali B Abdul Jabbar, Muhammad Ali, Muhammad Abdullah Arain, Sardar Shahmir B Chauhan, Saulat Fatimi

**Affiliations:** 1 Cardiothoracic Surgery, Aga Khan University, Karachi, PAK; 2 Ophthalmology, Johns Hopkins University, Baltimore, USA; 3 Surgery, Johns Hopkins University, Baltimore, USA; 4 Surgery, Aga Khan University, Karachi, PAK

**Keywords:** zoonotic larval infection, cysts, parasitic tapeworm, echinococcosis, superior vena cava syndrome, hydatid cyst, lung, thorax

## Abstract

Hydatid cyst disease, caused by *Echinococcus* species, can present with a variety of symptoms depending on the location in the body. In this report, we present a rare case of a 25-year-old male with a hydatid cyst who presented with progressive dyspnea and swelling of the facial, neck, and arm veins, i.e., the symptoms of superior vena cava syndrome. CT scan showed a huge cystic lesion on the right side of the thorax compressing the superior vena cava. The total size of the cyst was 183 X 209.5 X 333 mm, which is the largest collection of hydatid cysts reported in the thorax. A median sternotomy was performed, and numerous hydatid cysts were removed. The patient was placed on albendazole for 12 months and post-operative follow-ups up to three years showed no disease on chest x-ray.

## Introduction

Hydatid disease is caused by *Echinococcus* species, and Pakistan is considered an endemic region [1,2]. Hydatid disease of the thorax, which may or may not involve lung parenchyma, can either remain asymptomatic for many years or present with cough, chest pain, dyspnea, and hemoptysis, depending on the size of the cysts [1,3,4]. The cysts have a propensity to occur anywhere in the body; therefore, one of the primary manifestations of the cysts is the mechanical compression of the surrounding structures [4,5]. They are usually diagnosed using ultrasonography or CT scan. The treatment is either surgical removal of the cysts or medical therapy with benzimidazoles [3-6].

In this report, we present a case of superior vena cava (SVC) syndrome as a late and rare complication of the largest known collection of hydatid cysts in the thorax.

## Case presentation

**Figure 1:**
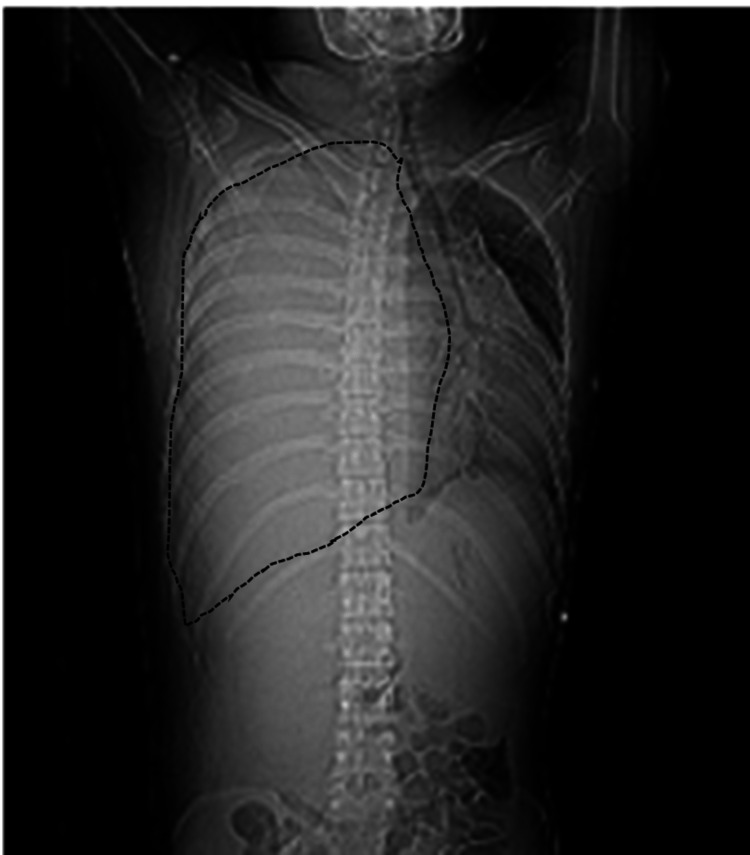
Chest x-ray (CXR) CXR Shows complete opacification of the right hemithorax with a marked leftward shifting of the trachea and the heart (black dotted line)

A 25-year-old gentleman from rural Sindh with a positive smoking history [three pack years] and no prior co-morbidities presented to the Emergency Room (ER) of Aga Khan University Hospital, Karachi, Pakistan. He reported pain in his right chest and hypochondrium for the past 45 days and shortness of breath at rest, which had progressively increased over the past five days. Severe orthopnea and a gradually increasing dry cough were also present. He had massive facial edema (facial plethora) but no pedal edema. His physical examination revealed reduced air entry in the entire right hemithorax. The patient came from a rural area of Sindh where healthcare is not widely accessible, Due to this, he waited until symptoms became unbearable. Upon his current presentation, the patient underwent a set of investigations. The chest x-ray (CXR) showed complete opacification of the right hemithorax and a marked leftward shift of the mediastinum (Figure 1).

**Figure 2:**
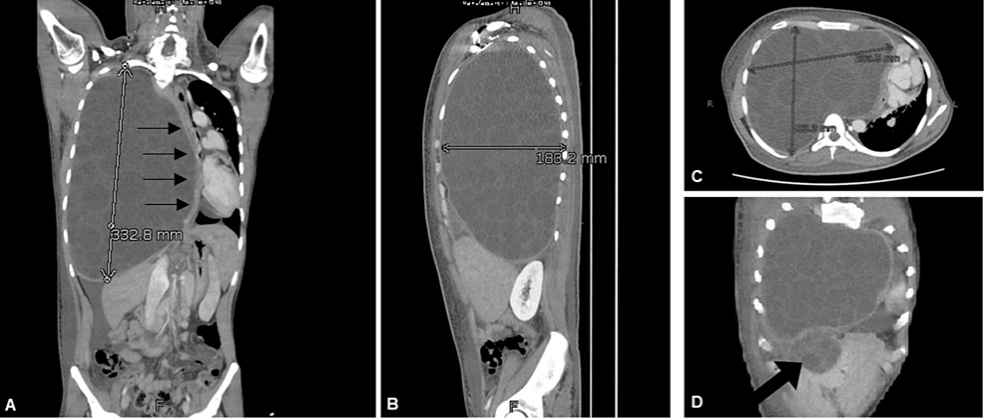
CT chest coronal sections (A and D), sagittal section (B), and axial sections (C) CT chest shows a huge hydatid cyst containing innumerable daughter cysts occupying the entire right hemithorax and crossing the midline with a significant cardio-mediastinal shift towards the left (thin black arrows in A) and significantly attenuated superior vena cava. The total size of the cysts measures 183 X 209.5 X 333 mm in maximum anteroposterior, transverse, and craniocaudal dimensions as shown in the images. (D) shows the exophytic cyst abutting the liver (thick black arrow).

A CT chest with intravenous contrast showed a large hypo-dense cystic lesion occupying the entire right hemithorax with right lung atelectasis, crossing the midline, resulting in a significant shift of the mediastinum towards the left side. Countless well-defined daughter cysts were also present. The SVC was significantly stretched out and attenuated by the cyst. The lesion measured 183 X 209.5 X 333 mm in maximum anteroposterior, transverse, and craniocaudal dimensions, respectively (Figure 2). Another exophytic hypo-dense cyst with peripheral calcifications, measuring 82 x 51 mm, was visualized just below the right hemidiaphragm, indenting the underlying liver (Figure 2D).

**Figure 3:**
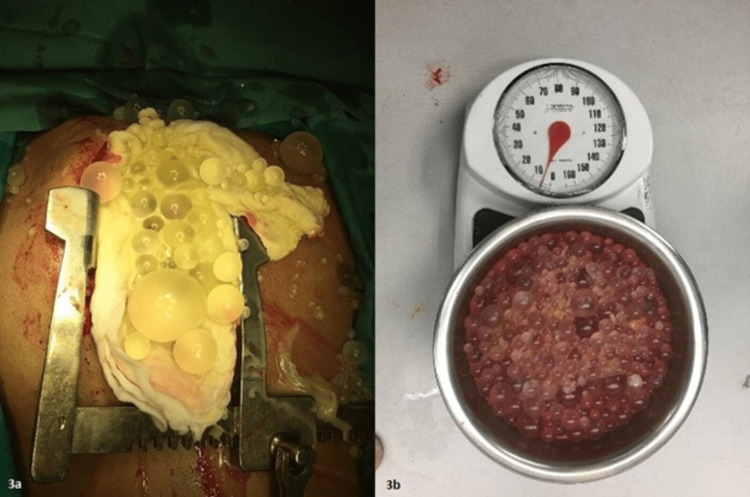
Gross appearance of cysts (3a) Intraoperative image: median sternotomy with a retractor covered by an adsorbent sponge; (3b) Weighing the collected hydatid cysts: approximately 5 kgs (11 lbs)

**Figure 4:**
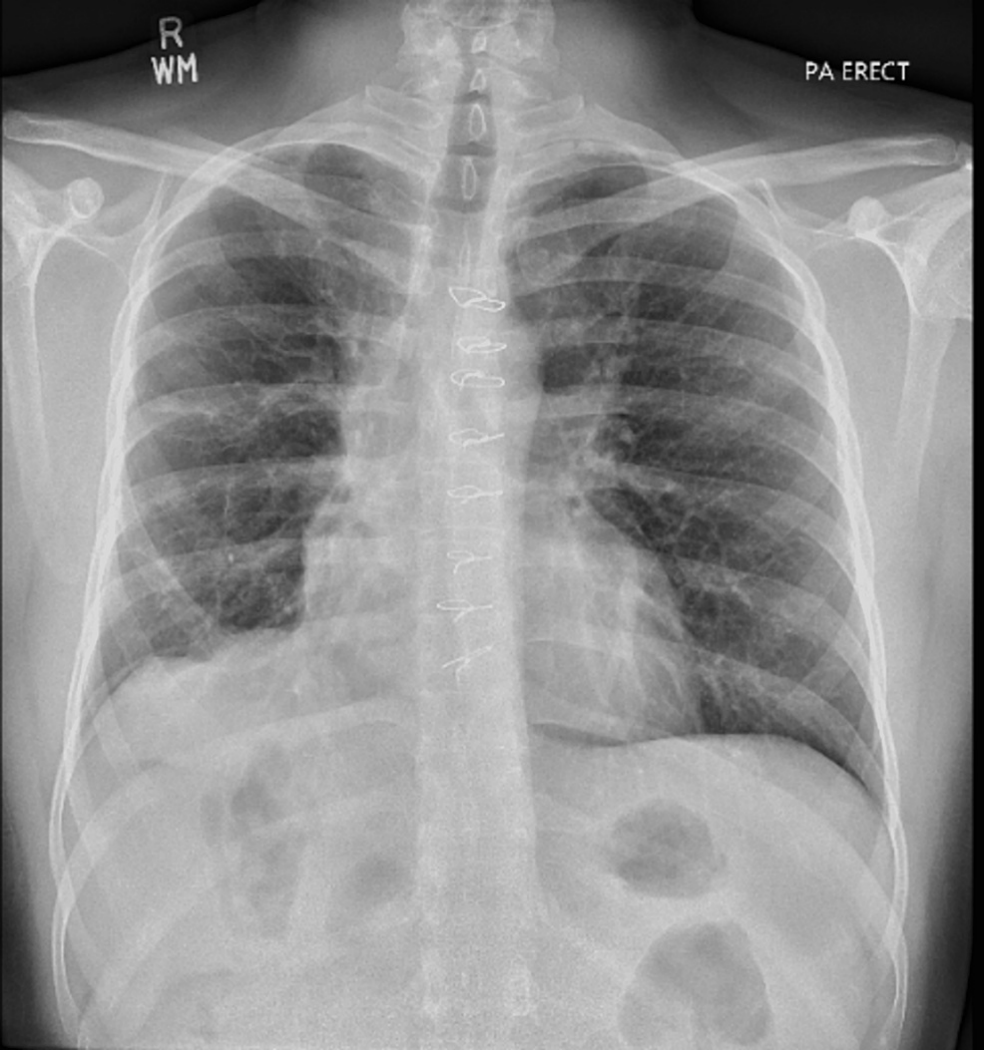
Post-operative follow-up chest x-ray (CXR) This CXR is three years after surgery and showed no disease recurrence

The patient underwent a median sternotomy with the removal of innumerable hydatid cysts from the right hemithorax without any lung resection (Figure 3). The symptoms of SVC syndrome resolved the moment the thorax was decompressed. The total weight of the cysts was above 5 kgs (Figure 3b). After removing the cyst, the right lung was found to be encased with a thick cortex of the cysts, which was removed in the surgery and the lung re-expanded. Three 32-French pleural drains were placed in the right thoracic cavity. During the procedure, the patient was placed on inotropic support and was weaned off the next day along with successful extubation. Five days later, he was able to fully ambulate and tolerate a regular diet and was deemed stable enough to be discharged home with an intrapleural chest tube on suction, which was later removed in the clinic. The patient was prescribed oral albendazole for 12 months, and postoperative follow-ups up to three years showed a re-expanded lung with no disease on CXR (Figure 4).

## Discussion

Hydatid disease is caused by the parasitic cestodes of *Echinococcus* species with *Echinococcus granulosus *being the most common pathogen. The disease typically occurs due to faecal-oral transmission of the *Echinococcus* eggs via farm animals, with dogs being the definitive host and sheep acting as an intermediate host. Humans, while being mere accidental hosts, may end up suffering from cystic disease upon ingestion of the eggs [4-6].

Typical symptoms of intrathoracic hydatid disease include cough (53-62%), chest pain (49-91%), dyspnea (10-70%), and hemoptysis (12-21%) [3,4]. Our patient presented with progressively worsening cough, chest pain, and dyspnea secondary to the mass effect and tension physiology of the intrathoracic cysts. Other less common symptoms not seen in this case include nausea, vomiting, malaise, and thoracic deformation [6,7].

In addition to the typical manifestations of the hydatid disease, our patient had facial plethora along with distended neck and arm veins. There was no pedal edema. The constellation of symptoms represented the existence of SVC syndrome as a sequela of the enlarging thoracic hydatid cysts. SVC syndrome is a combination of symptoms that results from an obstruction of blood flow through the SVC either due to internal obstruction from a device or catheter-related thrombosis, direct invasion of tumour into SVC, or due to extrinsic compression from any mass in the superior mediastinum. Typical symptoms of SVC syndrome include swelling of the face and neck with dyspnea and chest pain [8]. SVC compression secondary to intrathoracic hydatid cysts is extremely rare. Scientific literature has some evidence of cardiac hydatidosis presenting as SVC syndrome [7]; however, our extensive literature search did not yield a case where the hydatid disease of the pleura, lung, or mediastinum resulted in the typical presentation of SVC obstruction, rendering this presentation unique. 

Our patient presented with an amalgam of cysts in the right hemithorax and another exotic hypo-dense cyst, just below the right hemidiaphragm, indenting the underlying liver and stretching the right and left hepatic veins (Figure 2). The liver is the most common site of echinococcal hydatid cyst formation, harbouring close to 60% of the reported cases [4,5]. Lungs account for 20-30% of cases, making them the second most common site of cyst formation [4,5]. These cysts have a propensity to occur almost anywhere in the body and the rarer sites include the brain, kidneys, heart, muscles, and even bones [4-6].

A giant pulmonary hydatid cyst is defined as a cyst size greater than 100 mm (10 cm) [9,10]. The combined size of our patient’s cysts measured 183 X 209.5 X 333 mm in maximum anteroposterior, transverse, and craniocaudal dimensions, which, upon our extensive literature search, turns out to be the largest reported collection of cysts in the thoracic cavity. A previously reported pulmonary hydatid cyst that measured 150x 145 x 160 mm was described as “huge" [11]. This may indicate that, if left untreated, hydatid cyst disease could continue to expand en masse, resulting in an array of mechanical complications.

Surgery is considered the mainstay of treatment for the removal of hydatid cysts. Albendazole is started up to four days before the surgery and continued for over six months, as is the standard of care keeping in view surgical resection may cause unwanted contamination of the surrounding tissues [4,5]. In our case, medial sternotomy was performed as an emergency procedure to save a patient's life, in preference over lateral thoracotomy, because on the CT scan, it was not certain whether the disease was primarily in the lung, pleural cavity, or mediastinum. Interestingly during surgery, the disease was found in the mediastinum and pleural cavity of the right lung, forming a thick cortex covering the pleural cavity but sparing the lung parenchyma. Therefore, lung resection was not needed.

## Conclusions

Untreated hydatid disease could potentially lead to mechanical complications due to the mass effect of the cyst. The symptoms depend on the location of the cysts and the adjacent structures. Our case highlights that an untreated intrathoracic hydatid disease may progress to compress mediastinal structures including the SVC. Therefore, when suspected, prompt investigation and treatment of the disease may prevent rare complications such as the SVC syndrome.
